# Common genetic polymorphisms of microRNA biogenesis pathway genes and breast cancer survival

**DOI:** 10.1186/1471-2407-12-195

**Published:** 2012-05-28

**Authors:** Hyuna Sung, Sujee Jeon, Kyoung-Mu Lee, Sohee Han, Minkyo Song, Ji-Yeob Choi, Sue K Park, Keun-Young Yoo, Dong-Young Noh, Sei-Hyun Ahn, Daehee Kang

**Affiliations:** 1 Department of Biomedical Sciences, Seoul National University College of Medicine, Seoul, South Korea; 2 Department of Molecular Medicine and Biopharmaceutical Sciences, Graduate School of Convergence Science and Technology and College of Medicine or College of Pharmacy, Seoul National University, Seoul, South Korea; 3 Department of Environmental Health, Korea National Open University, Seoul, South Korea; 4 Department of Preventive Medicine, Seoul National University College of Medicine, Seoul, South Korea; 5 Cancer Research Institute, Seoul National University College of Medicine, Seoul, South Korea; 6 Department of Surgery, Seoul National University College of Medicine, Seoul, South Korea; 7 Department of Surgery, University of Ulsan College of Medicine and Asan Medical Center, Seoul, South Korea

**Keywords:** microRNA biogenesis pathway, Breast cancer, Survival, Single nucleotide polymorphism

## Abstract

**Background:**

Although the role of microRNA’s (miRNA’s) biogenesis pathway genes in cancer development and progression has been well established, the association between genetic variants of this pathway genes and breast cancer survival is still unknown.

**Methods:**

We used genotype data available from a previously conducted case–control study to investigate association between common genetic variations in miRNA biogenesis pathway genes and breast cancer survival. We investigated the possible associations between 41 germ-line single-nucleotide polymorphisms (SNPs) and both disease free survival (DFS) and overall survival (OS) among 488 breast cancer patients. During the median follow-up of 6.24 years, 90 cases developed disease progression and 48 cases died.

**Results:**

Seven SNPs were significantly associated with breast cancer survival. Two SNPs in *AGO2* (rs11786030 and rs2292779) and *DICER1* rs1057035 were associated with both DFS and OS. Two SNPs in *HIWI* (rs4759659 and rs11060845) and *DGCR8* rs9606250 were associated with DFS, while *DROSHA* rs874332 and *GEMIN4* rs4968104 were associated with only OS. The most significant association was observed in variant allele of *AGO2* rs11786030 with 2.62-fold increased risk of disease progression (95% confidence interval (CI), 1.41-4.88) and in minor allele homozygote of *AGO2* rs2292779 with 2.94-fold increased risk of death (95% CI, 1.52-5.69). We also found cumulative effects of SNPs on DFS and OS. Compared to the subjects carrying 0 to 2 high-risk genotypes, those carrying 3 or 4–6 high-risk genotypes had an increased risk of disease progression with a hazard ratio of 2.16 (95% CI, 1.18- 3.93) and 4.47 (95% CI, 2.45- 8.14), respectively (*P* for trend, 6.11E-07).

**Conclusions:**

Our results suggest that genetic variants in miRNA biogenesis pathway genes may be associated with breast cancer survival. Further studies in larger sample size and functional characterizations are warranted to validate these results.

## Background

Breast cancer is the most common cancer among females and represents 14% of female cancer deaths worldwide [1]. In Korea, breast cancer is the second most common cancer comprising 14.7% in year 2008 and the incidence rate has increased 6.8% annually during the last 6 years. The age-specific incidence rates of breast cancer among Korean women are somewhat different from those of western country, showing their highest peak in the 45–49 years age group with the proportion of young age-onset breast cancer much higher than in western countries [2-4]. While breast cancer incidence has increased over the past 30–40 years, mortality has remained stable or even decreased in the last 10–15 years, a likely result of earlier detection and improved treatment strategy. [5]. However, breast cancer survival can vary considerably not only among different ethnic groups but also across different subtypes of breast cancer. These differences have been partially explained by the traditional prognostic and predictive factors related to clinical and pathological features of breast cancer [6]. Previous studies conducting candidate gene approach and recently added genome-wide association studies have implicated inherited factors as influences on breast cancer survival. However, the reported effect size was very small to moderate and do not fully explain the heterogeneity in breast cancer survival [7-9].

MicroRNAs (miRNAs) are a major class of endogenous short non-coding RNAs that post-transcriptionally modulate gene expression in a sequence specific manner [10]. The role of miRNAs in human cancer pathogenesis has been well established by the identification of aberrant expression of miRNAs in many types of cancer including breast cancer [11]. It is also suggested that the perturbations in miRNA expression facilitate the key pathways involved in cancer progression such as inflammation, cancer cell invasion, angiogenesis, and metastasis [12]. Some miRNAs were reported to be associated with invasive and metastatic phenotype of breast cancer cell lines and to be also correlated with metastatic tumor tissues as well. On the other hand, some miRNAs serve as metastasis suppressors and it was shown that their expression is frequently downregulated or lost in both breast cancer cell lines and metastatic foci [13].

There is increasing evidence that the presence of genetic variants in precursor or mature miRNAs, their biogenesis pathway genes, or in miRNA binding sites of target mRNA are associated with the risk of cancer development [14]. More recently, the effects of genetic variants in miRNA related genes have been demonstrated to be associated with cancer survival in several types of cancers including colorectal cancer [15], renal cell carcinoma [16], ovarian cancer [17] and head and neck cancer [18].

Although the role of miRNA’s biogenesis pathway genes in cancer development and its progression has been well established, the association between genetic variants of this pathway genes and breast cancer survival is still unknown. Here, we selected 41 SNPs in 14 candidate genes (*AGO1, AGO2, DICER1, DGCR8, DROSHA, FMR1, GEMIN3, GEMIN4, HIWI, RAN, TARBP2, XPO5, p68* and *p72*) involved in the canonical miRNA biogenesis pathway [19,20] and evaluated the associations with breast cancer survival.

## Methods

### Study population

The REMARK criteria was used to report our data [21]. The study subjects were derived from the previous study to investigate association between the polymorphisms in miRNA biogenesis pathway genes and breast cancer risk [22]. The description of the Seoul Breast Cancer Study (SeBCS) has been described in the previous study. Briefly, histologically confirmed breast cancer cases (n = 3,497) and controls (n = 1,273) were recruited by the Seoul National University Hospital and Asan Medical Center between 2001 and 2007. A questionnaire was given by the trained interviewers to collect information on demographic, reproductive and other lifestyle factors. Peripheral blood was drawn into 10-mL heparinized tubes and stored at −70°C until genotyping. Those with previous history of cancer (n = 156), previous history of hysterectomy or oophorectomy (n = 268) were excluded. After the additional exclusion of the subjects with no or insufficient DNA samples (n = 760), 559 patients were available for genotyping experiment. Patients were followed up to March 2010 using a retrospective chart review with standard protocol to collect clinicopathological features, patients’ treatments, and vital status such as recurrence and death. The vital status was additionally checked through the death registry of the Korea National Statistical Office in May 2011.

Patients diagnosed with *in situ* breast cancer (n = 49), benign breast cancer (n = 13), or multiple cancer at diagnosis (n = 2) and with incomplete linkage of both clinical record and death record due to error in resident registration number (n = 7) were additionally excluded for survival analysis. Thus, a total of 488 invasive breast cancer patients were included in the final analysis. The baseline characteristics by study inclusion were presented in Additional file 1: Table S 1.

Informed consents were received from every patient when the questionnaire was administered. The study design was approved by the Committee on Human Research of Seoul National University Hospital (IRB No. H-0503-144-004).

### SNP selection and genotyping

The selection of genes and SNPs has been previously described in detail [22] . Using the data from the International HapMap Project (http://www.hapmap.org/), dbSNP (NCBI, http://www.ncbi.nlm.nih.gov/projects/SNP/) and web-based SNP selection tools (http://www.niehs.nih.gov/snpinfo) [23], we selected 41 haplotype tagging SNPs (htSNPs) defined by the linkage disequilibrium (LD) with square of the pairwise correlation coefficient, r^2^ > 0.90, in the genomic region from 5 kb upstream and 3 kb downstream of the largest cDNA isoform of each gene with minor allele frequency (MAF) of > 0.05 in Asian populations; 35 SNPs in 12 genes in miRNA biogenesis pathway and 6 SNPs in 2 genes involved in hormonal regulation of miRNA biogenesis. To measure the LD between SNPs, LD parameters (r^2^ and D′) were calculated using Haploview version 4.2 software (Whitehead Institute, Cambridge, MA, http://www.broadinstitute.org/haploview/haploview). In the case of multiple potentially functional SNPs within the same haplotype block, only one SNP was included in our analyses. The proportion of common variants tagged with r^2^ more than 0.8 ranged from 32.4% to 100% [22] .

Genotyping for 41 SNPs was conducted using the TaqMan assay (Applied Biosystems, Carlsbad, California). Primers and probes are available upon requests from the authors. Randomly selected 5% of samples and negative controls were included to ensure the accuracy of genotyping. The concordance rates for quality-control samples were >95.0% for all assays. For all 41 SNPs investigated, no significant deviation from Hardy-Weinberg equilibrium was observed among the controls (*P* > 0.01). We discarded one SNP in *TARBP* (rs2280448) that showed minor allele frequency less than 5%.

### Statistical analyses

The differences in patient characteristics by study inclusion were assessed by the Pearson’s *χ*^2^ tests for categorical variables and the Student's *t*-test for continuous variable.

The primary outcomes were disease-free survival (DFS) and overall survival (OS). For DFS analysis, we only included patients with stage I-III (*n* = 480) excluding patients diagnosed with stage IV (*n* = 8). DFS time was defined as the time from the date of surgery until the date of the first locoregional recurrence, first distant metastasis, 2^nd^ primary cancer or death from any cause. Patients known to be alive with no evidence of disease progression were censored at the last follow-up date or 31 March 2010, whichever came first. OS time was defined as the time from diagnosis until the date of death from any cause, censoring at the date of the last follow up or 31 May 2011, whichever came first.

Kaplan-Meier method was used to estimate the survival function, and differences across survival curves were examined using log-rank test. For the DFS analysis, Cox’s proportional hazard regression models were used to estimate hazard ratios (HR) and 95% confidence intervals (CI) adjusting for age at diagnosis, tumor size (≤ 2 cm and > 2 cm), lymph-node involvement (no and yes), histologic grade (I-II and III), nuclear stage (I-II and III), estrogen receptor (ER) status (positive and negative), progesterone receptor (PR) status (positive and negative), and hormone receptor therapy (yes and no) in the final model. For the OS analysis, tumor size (≤ 2 cm and > 2 cm), lymph-node involvement (no and yes), distal organ metastasis (no and yes), histologic grade (I-II and III) and hormone receptor therapy (yes and no) were included in the final model. Other covariates considered but not included the final model were menopausal status, adjuvant chemotherapy, and radiotherapy. Because these variables did not alter HRs significantly after adjusting for other covariates (statistical significance for the inclusion of the final model was set at *P* < 0.20) and the final model adjusted for all the covariates made no substantial difference to the results. The significance of the full versus reduced model was calculated with an F-test.

The proportional hazard assumption of the Cox model was examined by graphic evaluation of Schoenfeld residual plot. We generated dummy variables indicating the missing (1) and non-missing (0) for the all covariates and all the missing values of covariates were coded as ‘-1’. When we tested the regression model, each covariate was included in the model with respective dummy variable for missingness so that all the patients were included in the statistical models.

For individual SNP analysis, we tested three genetic models (additive, dominant and recessive) to evaluate the significance of SNPs and the best fitting model for each SNP was selected by the smallest *P* value.

Given the number of SNPs investigated, the Benjamini-Hochberg false discovery rate (FDR) method was used to assess the statistical significance after correction for multiple comparisons. We considered FDR < 0.05 as being noteworthy [24].

For haplotype analysis, the missing data with at least one of five polymorphic sites were excluded. Assuming HWE, the expectation-maximization algorithm was used to calculate the maximum likelihood estimates of the haplotype frequencies using SAS PROC HAPLOTYPE. The haplotypes with frequencies less than 5% in all patients were combined into one group. The most common haplotype was compared with other haplotypes with adjustment for selected covariates. Haplotype data were treated as categorical variable and were incorporated as dummy variables in the Cox model. Cumulative effects of SNPs were assessed by counting the number of high-risk genotypes in each subject. High-risk genotypes were defined as the genotypes with risk conferring-allele shown to be significantly associated with DFS or OS. The number of high-risk genotypes was categorized as low, medium, and high-risk groups and HRs and 95% CIs were calculated for all groups and compared with the low-risk group.

All statistical procedures were conducted using SAS version 9.2 (SAS Institute, Cary, NC). All *P* values reported were two-sided.

## Results

The differences between the patients’ characteristics by study inclusion were evaluated (Supplementary Table 1). All the clinical characteristics compared were not significantly different between two groups other than the distribution of age. The patients included in this study were younger than those excluded from the study (mean (standard deviation), 46.6 (11.0) *vs.* 47.9 (9.8), *P* = 0.02), however, the menopausal status were not significantly different between the groups.

**Table 1:** Baseline characteristics of subjects by follow up status

	Disease-free survival (n = 480)						Overall survival (n = 488)							
	Relapse (n = 90)^a^	No relapse (n = 389)	HR (95% CI)	*P*	aHR (95% CI) ^b^	*P*	Death (n = 41)	Alive (n = 447)	HR (95% CI)	*P*	aHR (95% CI) ^c^	*P*		
Follow up duration, years (median)	0.12-8.34 (4.64)						0.18-9.45 (6.24)					
Age (mean(SD))	46.3 (12.5)	46.6 (10.6)	0.99 (0.97- 1.01)	0.470	1.00 (0.98- 1.02)	0.918	45.8 (13.8)	46.8 (10.7)	0.98 (0.96- 1.01)	0.289	0.99 (0.97- 1.02)	0.632
Tumor size												
≤2 cm	27 (30.0)	210 (55.1)	1 (reference)		1 (reference)		10 (21.7)	229 (52.9)	1 (reference)		1 (reference)	
>2 cm	63 (70.0)	171 (44.9)	2.49 (1.59- 3.91)	<.001	1.66 (1.01- 2.72)	0.045	36 (78.3)	204 (47.1)	3.73 (1.85- 7.52)	<.001	2.20 (1.03- 4.67)	0.041
Lymph-node involvement												
No	36 (40.0)	258 (67.5)	1 (reference)		1 (reference)		15 (32.6)	282 (65.0)	1 (reference)		1 (reference)	
Yes	54 (60.0)	124 (32.5)	3.00 (1.97- 4.58)	<.001	2.54 (1.60- 4.03)	<.001	31 (67.4)	152 (35.0)	3.86 (2.08- 7.16)	<.001	2.70 (1.40- 5.20)	0.003
Metastasis												
No	90 (100)	389 (100)	-	-	-	-	42 (91.3)	432 (99.1)	1 (reference)		1 (reference)	
Yes							4 (8.7)	4 (0.9)	7.15 (2.55-20.02)	<.001	5.26 (1.71-16.15)	0.004
Histologic grade												
I-II	23 (30.7)	207 (61.4)	1 (reference)		1 (reference)		12 (31.6)	221 (58.3)	1 (reference)		1 (reference)	
III	52 (69.3)	130 (38.6)	3.00 (1.84- 4.90)	<.001	3.40 (1.74- 6.64)	<.001	26 (68.4)	158 (41.7)	2.81 (1.42- 5.56)	0.003	1.93 (0.93- 4.03)	0.078
Nuclear grade												
I-II	21 (40.4)	146 (59.4)	1 (reference)		1 (reference)		8 (34.8)	160 (57.1)	1 (reference)		1 (reference)	
III	31 (59.6)	100 (40.7)	1.82 (1.05- 3.17)	0.034	0.59 (0.28- 1.23)	0.161	15 (65.2)	120 (42.9)	2.52 (1.07- 5.94)	0.035	1.12 (0.40- 3.11)	0.830
Estrogen receptor												
Positive	49 (55.1)	238 (63.0)	1 (reference)		1 (reference)		22 (48.9)	269 (62.9)	1 (reference)		1 (reference)	
Negative	40 (44.9)	140 (37.0)	1.33 (0.88- 2.03)	0.177	1.69 (0.79- 3.62)	0.177	23 (51.1)	159 (37.2)	1.82 (1.02- 3.28)	0.044	1.02 (0.37- 2.80)	0.975
Progesterone receptor												
Positive	42 (47.7)	202 (53.6)	1 (reference)		1 (reference)		18 (40.0)	230 (54.0)	1 (reference)		1 (reference)	
Negative	46 (52.3)	175 (46.4)	1.23 (0.81- 1.87)	0.331	1.53 (0.79- 2.96)	0.202	27 (60.0)	196 (46.0)	1.75 (0.96- 3.18)	0.066	1.14 (0.48- 2.73)	0.765
Adjuvant chemotherapy												
Yes	78 (88.6)	285 (76.0)	1 (reference)		1 (reference)		41 (91.1)	327 (76.9)	1 (reference)		1 (reference)	
No	10 (11.4)	90 (24.0)	0.47 (0.24- 0.91)	0.026	1.00 (0.47- 2.14)	0.996	4 (8.9)	98 (23.1)	0.36 (0.13- 1.00)	0.049	1.16 (0.37- 3.60)	0.804
Hormone receptor therapy												
Yes	47 (52.8)	263 (70.1)	1 (reference)		1 (reference)		21 (46.7)	292 (68.5)	1 (reference)		1 (reference)	
No	42 (47.0)	112 (29.9)	1.79 (1.18- 2.71)	0.006	2.43 (1.17- 5.07)	0.018	24 (53.3)	134 (31.5)	2.33 (1.30- 4.18)	0.005	1.51 (0.80- 2.85)	0.201
Radiotherapy												
Yes	43 (48.9)	185 (49.7)	1 (reference)		1 (reference)		20 (43.5)	211 (50.0)	1 (reference)		1 (reference)	
No	45 (51.1)	187 (50.3)	0.90 (0.59- 1.37)	0.633	0.96 (0.62- 1.48)	0.843	26 (56.5)	211 (50.0)	1.10 (0.61- 1.97)	0.760	1.12 (0.61- 2.04)	0.723

NOTE Column frequency may be less than total number of subjects due to missing values.

^a^ Disease free survival event (relapse) included locoregional recurrence (n=76), 2nd primary cancer (n=11), and death (n=3) from any cause.

^b^ Adjusted for age, tumor size (≤2cm and >2cm), lymph-node involvement (no and yes), histologic grade (I-II and III), nuclear grade (I-II and III) and ER status (positive and negative), PR status (positive and negative) and hormone receptor therapy (yes and no.).

^c^ Adjusted for age, tumor size (≤2cm and >2cm), lymph-node involvement (no and yes), metastasis (no and yes), histologic grade (I-II and III), and hormone receptor therapy (yes and no).

Of the 488 invasive breast cancer patients included in this study, 323 (66.5%) patients were premenopausal women, 449 cases patients (92.0%) had ductal carcinoma, 37 cases (7.6%) had either lobular, mucinous or papillary carcinoma, and 2 cases were unknown. Over 83% of all cases (*n* = 408) had early stage disease (stage I-IIB).

During a median follow-up of 4.24 years (range, 0.1-8.3 years) of DFS, there were 76 recurrences, 11 second primary cancers, and 3 deaths among the 480 patients diagnosed with stage I-III. In addition, during a median follow-up of 6.24 years (range, 0.2-9.5 years) of OS, there were 41 deaths from any cause among the 488 patients. Table 1 summarized univariate and multivariate–adjusted HRs for DFS and OR by patients' characteristics. There were significant differences across survival curves of DFS and OS according to tumor size, lymph-node involvement, metastasis, histologic grade, nuclear grade, ER receptor, adjuvant chemotherapy and hormone receptor therapy (log-rank *P* < 0.05, data not shown). In multivariate analysis, tumor size, lymph-node involvement, histologic grade, nuclear grade, ER status, PR status, and hormone receptor therapy were remained as independent and significant prognostic factors for DFS and tumor size, lymph-node involvement, metastasis, histologic grade and hormone receptor therapy for OS (*P* < 0.20).

Results from association analyses for 40 SNPs and breast cancer prognosis are presented in Table2. There were seven SNPs significantly associated with breast cancer survival. Two SNPs in *AGO2* (rs11786030 and rs2292779) and *DICER1* rs1057035 were associated with both DFS and OS. Two SNPs in *HIWI* (rs4759659 and rs11060845) and *DGCR8* rs9606250 were associated with DFS, while *DROSHA* rs874332 and *GEMIN4* rs4968104 were associated with only OS (*P* < 0.05, Table 2). The statistical significance was retained after multiple comparisons only for the association between *AGO2* rs2292779 and OS.

**Table 2:** Association of SNPs in microRNA biogenesis pathway genes and breast cancer survival

Gene	SNP	Position	Major/Minor allele	MAF^a^	Disease-free survival				Overall survival			
					Genetic model	aHR (95 % CI) ^b^	*P*	FDR *P*	Genetic model	aHR (95 % CI) ^c^	*P*	FDR *P*
*AGO1*	rs595055	intron	G/A	0.17	REC	1.86 (0.65-5.30)	0.247	0.47	ADD	0.66 (0.36-1.21)	0.181	0.44
	rs11263833	intron	T/G	0.24	REC	1.35 (0.56-3.27)	0.500	0.61	REC	0.56 (0.13-2.38)	0.430	0.60
*AGO2*	rs2292779	intron	C/G	0.38	ADD	1.42 (1.06-1.92)	0.021	0.27	REC	2.94 (1.52-5.69)	0.001	0.05
	rs3864659	intron	A/C	0.05	ADD	0.85 (0.44-1.62)	0.619	0.69	DOM	1.11 (0.43-2.85)	0.825	0.93
	rs7016981	intron	T/C	0.10	ADD	0.77 (0.46-1.30)	0.326	0.49	DOM	1.48 (0.75-2.91)	0.254	0.50
	rs7824304	intron	C/T	0.05	ADD	0.53 (0.24-1.18)	0.120	0.36	ADD	0.59 (0.20-1.75)	0.340	0.57
	rs11786030	3’ UTR	A/G	0.48	DOM	2.62 (1.41-4.88)	0.002	0.09	DOM	2.41 (1.05-5.50)	0.037	0.38
*DGCR8*	rs2073779	intron	T/C	0.08	DOM	0.89 (0.50-1.60)	0.706	0.72	ADD	0.92 (0.43-1.97)	0.836	0.93
	rs9605062	intron	T/C	0.14	ADD	0.65 (0.40-1.06)	0.084	0.34	ADD	0.62 (0.30-1.26)	0.186	0.44
	rs9606250	intron	A/T	0.05	DOM	0.21 (0.05-0.84)	0.028	0.28	-	-	-	0.99
*DICER1*	rs1057035	3’ UTR	T/C	0.09	DOM	1.72 (0.99-2.99)	0.054	0.31	DOM	2.08 (1.01-4.28)	0.047	0.38
	rs2282265	intron	A/G	0.45	REC	1.70 (0.99-2.91)	0.055	0.31	DOM	0.87 (0.44-1.74)	0.700	0.85
*DROSHA*	rs644236	intron	C/T	0.31	REC	0.80 (0.41-1.58)	0.517	0.61	ADD	0.73 (0.46-1.15)	0.173	0.44
	rs874332	intron	T/C	0.47	REC	1.25 (0.76-2.05)	0.382	0.54	REC	2.24 (1.21-4.17)	0.011	0.21
	rs7737174	intron	G/A	0.46	REC	1.13 (0.69-1.85)	0.639	0.69	REC	0.76 (0.36-1.61)	0.469	0.60
	rs10461898	intron	A/G	0.23	REC	1.75 (0.68-4.46)	0.244	0.47	ADD	1.44 (0.86-2.41)	0.170	0.44
	rs11748548	intron	G/A	0.26	DOM	0.79 (0.51-1.24)	0.307	0.49	ADD	0.71 (0.43-1.16)	0.167	0.44
	rs16901096	intron	C/T	0.21	DOM	0.73 (0.46-1.16)	0.185	0.44	DOM	0.58 (0.30-1.11)	0.098	0.44
*FMR1*	rs25704	3’ UTR	T/C	0.35	DOM	1.27 (0.82-1.97)	0.287	0.49	DOM	1.39 (0.74-2.61)	0.302	0.52
	rs28900	intron	A/C	0.46	DOM	1.21 (0.75-1.94)	0.443	0.59	DOM	1.63 (0.81-3.29)	0.175	0.44
	rs971000	intron	C/T	0.33	DOM	1.45 (0.94-2.23)	0.095	0.35	DOM	1.71 (0.92-3.18)	0.089	0.44
*GEMIN3*	rs197413	V642V	G/A	0.39	REC	1.33 (0.78-2.26)	0.299	0.49	REC	0.65 (0.25-1.66)	0.368	0.58
	rs17569368	intron	A/T	0.09	DOM	1.38 (0.80-2.37)	0.242	0.47	DOM	1.14 (0.54-2.42)	0.727	0.86
*GEMIN4*	rs7813	C1033R	T/C	0.35	ADD	0.77 (0.56-1.07)	0.126	0.36	REC	0.69 (0.27-1.82)	0.459	0.60
	rs2740349	N929D	A/G	0.13	DOM	0.83 (0.47-1.44)	0.502	0.61	DOM	0.81 (0.37-1.79)	0.604	0.76
	rs3744741	Q684R	C/T	0.22	REC	1.21 (0.52-2.81)	0.662	0.7	REC	1.83 (0.64-5.28)	0.262	0.50
	rs4968104	E593V	T/A	0.15	DOM	0.62 (0.36-1.06)	0.078	0.34	ADD	0.46 (0.21-0.99)	0.048	0.38
*HIWI*	rs4759659	intron	G/A	0.14	ADD	0.50 (0.29-0.85)	0.011	0.22	ADD	0.67 (0.32-1.39)	0.281	0.51
	rs7963072	intron	G/A	0.1	REC	1.71 (0.23-12.7)	0.598	0.68	REC	4.30 (0.57-32.5)	0.158	0.44
	rs1106042	K527R	G/A	0.11	DOM	1.05 (0.61-1.82)	0.859	0.86	DOM	1.04 (0.50-2.16)	0.908	0.95
	rs11060845	intron	G/T	0.4	ADD	1.36 (1.00-1.85)	0.054	0.31	DOM	1.76 (0.88-3.54)	0.110	0.44
*p68*	rs1991401	5’ UTR	A/G	0.36	ADD	0.87 (0.63-1.20)	0.391	0.54	REC	1.35 (0.60-3.07)	0.467	0.60
*p72*	rs138457	intron	T/C	0.5	ADD	1.19 (0.87-1.63)	0.274	0.49	DOM	1.80 (0.83-3.90)	0.134	0.44
	rs138462	3’ UTR	A/G	0.12	REC	1.60 (0.38-6.69)	0.519	0.61	DOM	0.72 (0.35-1.48)	0.378	0.58
	rs12627803	intron	T/C	0.21	REC	0.53 (0.15-1.91)	0.329	0.49	DOM	1.27 (0.69-2.33)	0.446	0.60
	rs2267390	intron	C/T	0.37	REC	0.58 (0.27-1.23)	0.156	0.42	REC	0.45 (0.14-1.47)	0.186	0.44
	rs5750609	intron	C/T	0.16	REC	0.28 (0.04-2.06)	0.213	0.47	ADD	0.69 (0.37-1.28)	0.245	0.50
*RAN*	rs7958223	intron	C/A	0.2	DOM	1.35 (0.88-2.07)	0.176	0.44	REC	2.02 (0.60-6.78)	0.257	0.50
	rs10848236	intron	G/A	0.16	DOM	1.52 (0.98-2.37)	0.062	0.31	ADD	1.03 (0.58-1.84)	0.909	0.95
*XPO5*	rs11077	3’ UTR	A/C	0.09	ADD	0.56 (0.27-1.18)	0.128	0.36	ADD	0.96 (0.44-2.09)	0.922	0.95

NOTE ADD, additive model; DOM, dominant model; REC, recessive model.

^a^ Major/minor alleles and frequencies as determined by the distribution among all patients.

^b^ Adjusted for age, tumor size (≤2 cm and >2 cm), lymph-node involvement (no and yes), histologic grade (I-II and III), nuclear grade (I-II and III) and ER status (positive and negative), PR status (positive and negative) and hormone receptor therapy (yes and no).

^c^ Adjusted for age, tumor size (≤2 cm and >2 cm), lymph-node involvement (no and yes), metastasis (no and yes), histologic grade (I-II and III), and hormone receptor therapy (yes and no).

The most significant association was observed for the *AGO2* rs11786030 G allele. The cases with AG/GG genotypes had 2.62-fold increased risk of disease progression of breast cancer (95% confidence interval (CI), 1.41-4.88) and 2.41-fold increased risk of death (95% CI, 1.05-5.50). The *AGO2* rs2292779 was also associated with poor prognosis of breast cancer. The minor allele (G) of SNP rs2292779 was associated with 1.42-fold increased risk of disease progression in dose dependent manner (95% CI, 1.05-1.87) and the association with the risk of death was stronger with an adjusted HR of 2.94 in recessive model (95% CI, 1.18-4.35). In addition, the SNP rs1057035 located in 3’UTR of *DICER1* was also associated with both DFS and OS. The TC/CC genotypes of rs1057035 were associated with 1.72-fold increased risk of disease progression (95% CI, 1.00-2.99) and 2.08-fold increased the risk of death.

The *DGCR8* rs9606250 and two SNPs in *HIWI* (rs4759659 and 11060845) were associated with only DFS. The variant allele of *DGCR8* rs9606250 variant allele (T) was significantly associated DFS with an adjusted HR of 0.21 (95% CI, 0.05-0.84) in dominant model. The *HIWI* rs4759659 variant allele (A) was associated with decreased risk of disease progression (per-allele HR, 0.50; 95% CI, 0.29-0.85), however, rs11060845 variant allele (T) was associated with increased risk of disease progression.

The *DROSHA* rs874332 C allele was associated with increased risk of death in recessive model (adjusted HR _TT/TC *vs* CC_, 2.24; 95% CI, 1.21-4.17) and *GEMIN4* rs4968104 A allele was associated with decreased risk of death in dose dependent manner (per-allele HR, 0.46; 95% CI, 0.21-0.99).

We conducted haplotype analysis for 13 genes other than p68 and *XPO5*. We found that common haplotypes of AGO2 were associated with both DFS and OS (Table 3). The four most common haplotypes were G-A-T-C-A, C-A-T-C-G, G-A-T-C-G, and G-A-C-C-A with the respective frequencies of 37.1%, 26.6%, 19.6% and 3.5% accounting for 87.1% in all patients. We found the haplotype of G-A-T-C-G were significantly increased risk of disease progression and death compared to the most common haplotype of G-A-T-C-A (adjusted HR, 2.66; 95% CI, 1.49-4.73; *P* = 0.001 and adjusted HR, 3.11; 95% CI, 1.34-7.23; *P* = 0.008, respectively). No significant associations were observed with haplotype of other genes (data not shown).

**Table 3:** **Association of*AGO2*haplotypes and breast cancer survival**

		Disease-free survival					Overall survival	
Haplotype	Relapse, *N* (%)	No relapse, *N* (%)	aHR (95 % CI) ^a^	*P*	Death, *N* (%)	Alive, *N* (%)	aHR (95 % CI) ^b^	*P*
G-A-T-C-A	22 (25.9)	152 (39.9)	1 (reference)		9 (20.9)	167 (38.8)	1 (reference)	
C-A-T-C-G	24 (28.2)	100 (26.3)	1.29 (0.71-2.34)	0.411	13 (30.2)	113 (26.2)	1.73 (0.72-4.13)	0.217
G-A-T-C-G	27 (31.8)	63(16.5)	2.66 (1.49-4.73)	0.001	15 (34.9)	78 (18.1)	3.11 (1.34-7.23)	0.008
G-A-C-C-A	5 (5.9)	12 (3.2)	1.76 (0.66-4.71)	0.262	2 (4.65)	16 (3.7)	2.22 (0.47-10.6)	0.316
Others	7 (8.24)	54 (14.2)	0.66 (0.28-1.56)	0.343	4 (9.3)	57 (13.2)	1.02 (0.31-3.33)	0.978
Total	85 (100)	381 (100)			43 (100)	431 (100)		

NOTE The *AGO2* haplotype was composed of five SNPs in order of rs2292779, rs3864659, rs7016981, rs7824304, rs11786030.

^a^ Adjusted for age, tumor size (≤2 cm and >2 cm), lymph-node involvement (no and yes), histologic grade (I-II and III), nuclear grade (I-II and III) and ER status (positive and negative), PR status (positive and negative) and hormone receptor therapy (yes and no).

^b^ Adjusted for age, tumor size (≤2 cm and >2 cm), lymph-node metastasis (no and yes), metastasis (no and yes), histologic grade (I-II and III), and hormone receptor therapy (yes and no).

We also found cumulative effects of SNPs on DFS and OS. Compared with subjects carrying 0 to 2 high-risk genotypes, those carrying 3 and 4–6 high-risk genotypes had an increased risk of disease progression with an adjusted HR of 2.16 (95% CI. 1.18-3.93) and 4.47 (95% CI, 2.45-8.14), respectively (HR for trend, 2.11; *P* for trend, 6.11E-07). Similar pattern was observed as for OS, although the association was stronger than that for DFS (HR for trend, 2.80; *P* for trend, 3.30E-05) (Table 4). The Kaplan-Meier survival function is consistent with the result of proportional hazard assumption (data not shown).

**Table 4:** Cumulative effect analysis by the number of unfavorable genotypes in miRNA biogenesis pathway genes and disease free survival

Number of unfavorable genotypes	Disease-free survival			
	Cases, *N*^a^ (%)	Relapse, *N* (%)	aHR^b^ (95 % CI)	*P*
0-2	163 (35.4)	18 (21.4)	1 (reference)	
3	188 (40.9)	32 (38.1)	2.16(1.18- 3.93)	1.22E-02
4-6	109 (23.7)	34 (40.5)	4.47(2.45- 8.14)	9.87E-07
*P*_trend_			2.11(1.57- 2.83)	6.11E-07
	**Overall survival**			
	**Cases, *N***^**a**^** (%)**	**Death, *N* (%)**	**aHR**^**c**^**(95 % CI)**	***P***
0-2	6 (14.6)	124 (26.4)	1 (reference)	
3	12 (29.3)	214 (45.5)	1.21(0.44- 3.30)	7.13E-01
4-5	23 (56.1)	132 (28.1)	5.34(2.12-13.49)	3.93E-04
*P*_trend_			2.80(1.72- 4.55)	3.30E-05

NOTE Unfavorable genotypes were defined as rs2292779 (GG), rs11786030 (AG + GG), rs9606250 (AA + AT), rs1057035 (TC + CC), rs4759659 (GG + GA), and rs11060845 (TT) for disease-free survival and rs2292779 (GG), rs11786030 (AG + GG), rs1057035 (TC + CC), rs874332 (CC), and rs4968104 (TT) for overall survival, respectively.

^a^ Due to excluding the subject which has at least one missing genotype information in this analysis, all subjects are less than 480 for disease-free survival and 488 for overall survival.

^b^ Adjusted for age, tumor size (≤2 cm and >2 cm), lymph-node involvement (no and yes), histologic grade (I-II and III), nuclear grade (I-II and III) and ER status (positive and negative), PR status (positive and negative) and hormone receptor therapy (yes and no).

^c^ Adjusted for age, tumor size (≤2 cm and >2 cm), lymph-node involvement (no and yes), metastasis (no and yes), histologic grade (I-II and III), and hormone receptor therapy (yes and no).

## Discussion

In this study, we found seven SNPs significantly associated with breast cancer prognosis and gene-dosage effect of increasing number of high-risk genotypes on DFS and OS of breast cancer. Two SNPs in *AGO2* (rs11786030 and rs2292779) and *DICER1* rs1057035 were associated with both DFS and OS. Two SNPs in *HIWI* (rs4759659 and rs11060845) and *DGCR8* rs9606250 were associated with DFS, while *DROSHA* rs874332 and *GEMIN4* rs4968104 were associated with only OS. Furthermore, some specific *AGO2* haplotype also associated with both DFS and OS.

The *AGO2* plays a key role in miRNA-mediated gene silencing as a component of the miRNA-induced silencing complex that directly binds miRNAs and mediates cleavage of target mRNA [25]. There are emerging evidence from *in vitro* analysis and clinical samples that abnormal expression or enzymatic function of *AGO2* is associated with cancer development and its progression. In breast cancer cell lines, it was shown that the overexpression of *AGO2* could induce the transformed phenotype [26]. Moreover, the variations in genomic structure of *AGO2,* such as copy number change or frameshift mutation, were reported to be associated with several cancers including multiple myeloma, gastric and colorectal cancer [27,28].

We observed that variant alleles of rs2292779 and rs11786030 in *AGO2* were commonly associated with poor DFS and poor OS. In our previous study, we reported SNP rs3864659 in *AGO2* is associated with the reduction of breast cancer risk. However, we did not observe the significant association with breast cancer survival for rs3864659 implying its different role in tumor development and survival in breast cancer patients. The rs2292779 G allele was more significantly and strongly associated with OS than DFS, while the effect sizes of rs11786030 were similar between OS and DFS. Furthermore, the haplotype containing minor alleles of both SNPs (rs2292779 and rs11786030) was also significantly associated with both DFS and OS showing slightly larger effect than those of estimated from single SNP. The *AGO2* rs2292778 was not genotyped in our study, but it is a perfect proxy for *AGO1* rs2292779, based on the HapMap Chinese in Beijing (CHB) (r^2^ = 0.94) and Japanese in Tokyo (JPT) (r^2^ = 0.95) and even in CEPH European ancestry (CEU) (r^2^ = 1.00). It is intriguing that the *AGO2* rs2292778 is highly evolutionary conserved allele (conservation score = 0.992) although it resides outside the coding region of *AGO2* suggesting potential functional effect of these variants [25]. However, it should be answered whether the function or expression level of *AGO2* is regulated via proxy or identified SNP through experimental study.

The SNP rs9606250 in *DGCR8* was found to be associated with longer DFS. As for the association with OS, however, we could not evaluate the effect of rs9606250 on OS due to low frequency of minor allele homozygote. *DGCR8 (Pasha)* is a double stranded RNA-binding protein involved in processing of primary precursor of miRNAs into the pre-miRNAs as a component of multi-protein complex with RNAse III enzyme *DROSHA (RNASEN)*. The possible role of *DGCR8* gene in the clinical outcome of breast cancer has been recently reported [25]. It was shown that impaired miRNA processing through knockdown of *DGCR8* facilitates breast cancer cell invasion. Interestingly, both rs9605062 and rs9606250 identified in *DGCR8* is in high LD with rs9606232 (CHB and JPT, r^2^ = 1.00; CEU, r^2^ = 0.83), which is located at a conserved transcription-binding site of *DGCR8*. Thus, it is plausible that the level or the timing of gene expression might be regulated by these variants, although more detailed functional studies should provide evidence for the underlying mechanism. In previous studies, three SNPs in *DGCR8* were associated with OS of ovarian cancer consistent with our result [17], although there is no significant association between the genetic variants in *DGCR8* and survival of patients with colorectal cancer [15], head and neck cancer [18] and renal cell carcinoma [16].

We also observed the variant allele of *HIWI* rs4759659 was also associated with longer DFS, although it was not associated with OS. In addition, *HIWI* rs11060845 was associated with increased risk of disease progression in the present study, while this SNP was associated with risk reduction in breast cancer susceptibility in our previous study implicating the difference effect of the same variant on cancer development and progression. *HIWI* (*PIWI1*) is suggested to play an important role in stem cell renewal, division, germ cell proliferation, RNA silencing and translational regulation [29]. In addition, *HIWI* expression has been shown to be associated with tumor development including gastric cancer [30] and pancreas adenocarcinoma [31]. Furthermore, the expression level of *HIWI* has been shown to be associated with cancer survival in esophageal squamous cancer cells [32] and soft-tissue sarcoma [21]. It is notable that *HIWI* is significantly overexpressed in several metastatic tumor tissues compared to benign hyperplasia or chronic inflammation lesion, and the expression is positively correlated with Ang-2 and Tie-2 which play a key role in angiogenesis [33]. However, further study is warranted to prove whether the identified variant leads to decreased expression of *HIWI* gene and whether the effect of this variant on breast cancer microinvasion or metastasis is mediated by angiogenic pathway.

We also found the variant allele of *DROSHA* rs874332 was associated with poor OS in breast cancer. *DROSHA* (*RNASEN*) is RNAse III enzyme mediating processing of pri-miRNAs into pre-miRNAs with *DGCR8*. The significance of aberrant expression levels of *DROSHA* as a potential prognostic factor has been substantiated in several studies on esophageal cancer [34], cervical neoplastic progression [35], ovarian cancer [36], and neuroblastoma [37] .

In breast cancer, down regulation in *DROSHA* and/or *DICER* was preferentially observed in distinct subgroups of breast cancer [38] . In addition, the haplotypes of *DROSHA* and *DICER*, but not individual SNP, were associated with altered survival and recurrence of renal cancer carcinoma [16]. Through *in vitro* functional study, Noh *et al.* demonstrated that less efficient miRNA processing caused by knockdown of *DROSHADICER* and *DGCR8* is responsible for reduced levels of mature forms of tumor-suppressive miRNAs, and finally facilitates breast cancer cell invasion by upregulating the expression of urokinase-type plasminogen activator (uPA) [39]. Considering that *DROSHA* rs642321, the proxy of rs874332 (CHB r^2^ = 0.93; JPT, r^2^ = 0.87 and CEU, r^2^ = 0.82), is located in the 3’-untranslated region (UTR) of *DROSHA* and is a predicted miRNA binding site (http://www.microrna.org/microrna/home.do), it is plausible that rs874332 could be associated with translational repression and/or mRNA destabilization of *DROSHA* through miRNA-mRNA interaction.

We performed polygenic risk model analysis to investigate the cumulative effects of SNPs on breast cancer survival. By combining six SNPs for DFS and five SNPs for OS, we observed a strong dose–response trend toward an increasing risk of disease progression or death with an increasing number of high-risk genotypes. This cumulative effect of variants in miRNA biogenesis pathway genes on breast cancer survival is consistent with the notion that the germline genetic variation could be associated with cancer progression and survival via polygenic manner.

Although the present study included tagging SNPs in most currently known miRNA biogenesis pathway genes, future studies should investigate the comprehensive list of genetic variants in miRNA genes and target binding site in 3’-untranslated region to understand how their deregulation of miRNA network caused potentially by the genetic variants is implicated in breast cancer prognosis.

There are several limitations in this study. We could not evaluate the association between the selected SNPs and breast cancer specific mortality because the data for the cause of death were not available. Thus, the results on the association with the OS must be interpreted cautiously and need to be confirmed in study to investigate the association with breast cancer specific survival. The primary limitation is the limited power to detect the potential effects of genetic variants on the breast cancer prognosis, especially for the several SNPs with low minor allele frequencies. This study has only 1 to 66% of the statistical power to detect the effect sizes of 1.10 to 1.40 with the current sample size (Bonferroni corrected *p*-value = 0.001). Along with the limited power, some of the SNPs we identified might be attained by chance finding given the loss of robustness after multiple comparison adjustments. Furthermore, the risk profiles of genetic variants identified from this study could be manifested differently in different ethnic populations assuming the differences in MAF and underlying genomic structure, and interactions of environmental factors and other modifiers could have an effect on the penetrance of these SNPs.

## Conclusions

We evaluated the comprehensive list of tagging SNPs in most currently known miRNA biogenesis pathway genes and we identified the associations between several putative genetic variants in miRNA biogenesis pathway genes and breast cancer survival. To the best of our knowledge, this is the first study to provide evidence suggesting that common genetic variants in miRNA biogenesis pathway genes may affect the breast cancer survival, individually and jointly. To more powerfully elucidate this risk-conferring effect of any SNP observed in this study, further epidemiological studies in independent and large number of subjects and functional studies need to validate our findings.

## Abbreviations

BMI, Body mass index; CEU, CEPH European ancestry; CHB, Han Chinese in Beijing, China; CI, Confidence interval; DFS, Disease-free survival; ER, Estrogen receptor-positive; ER, Estrogen receptor-negative; GWA study, Genome-wide association study; HWE, Hardy-Weinberg equilibrium; JPT, Japanese in Tokyo; LD, Linkage disequilibrium; MAF, Minor allele frequency; MiRNA, microRNA; HR, Hazard ratio; OS, Overall survival; PR, Progesterone receptor-positive; PR, Progesterone receptor-negative; SNP, Single nucleotide polymorphism.

## Competing interests

The authors have declared no conflicts of interest.

## Authors’ contributions

DK, DYN, and SHA were PIs for each of the participating cooperative groups of the Seoul Breast Cancer Study (SeBCS). KML, JYC, SKP, KYY and DK were involved in conception and design of the study and participated in the discussion and interpretation of the results. HS participated in data analysis and writing of the manuscript. SJ, SH, MS and JYC contributed to statistical analyses and helped to draft the manuscript. SJ participated in data collection and sample selection. All authors have read and approved the final manuscript.

## Pre-publication history

The pre-publication history for this paper can be accessed here:

http://www.biomedcentral.com/1471-2407/12/195/prepub

## Supplementary Material

Additional file 1 **Table S1 **The baseline characteristics of the patients by study inclusion. Click here for file
