# Supplementary material for: Common genetic polymorphisms of microRNA biogenesis pathway genes and breast cancer survival
Source: BMC Cancer. 2012 May 28;12:195. doi: 10.1186/1471-2407-12-195 (PMC3487887; doi:10.1186/1471-2407-12-195)
Supplement: Additional file 1 — Table S1 The baseline characteristics of the patients by study inclusion. [file 1471-2407-12-195-S1.pdf]

Supplementary Table 1. The baseline characteristics of the patients by study inclusion.

	Included (*N* = 488)	Excluded (*N* = 2,327) [a]	*P* [b]
Age at diagnosis (mean(SD))	46.6 (11.0)	47.9 (9.8)	0.02[c]
Menopausal status			
Premenopausal	323 (66.5)	1489 (64.6)	0.47
Postmenopausal	163 (33.5)	816 (35.4)	
TNM stage			
IA-IB	182 (38.2)	817 (37.6)	0.69
IIA -IIB	226 (47.4)	1010 (46.4)	
IIIA - IV	69 (14.5)	349 (16.0)	
Tumor size			
≤2cm	239 (49.9)	1028 (45.8)	0.11
2cm<	240 (50.1)	1215 (54.2)	
Lymph-node involvement			
No	297 (61.9)	1303 (57.6)	0.08
Yes	183 (39.1)	959 (42.4)	
Metastasis			
No	474 (98.3)	2113 (98.2)	0.87
Yes	8 (1.7)	38 (1.8)	
Histologic grade			
I-II	233 (55.9)	1085 (55.5)	0.89
III	184 (44.1)	870 (44.5)	
Nuclear grade			
I-II			
III			
Estrogen receptor	291 (61.5)	1387 (62.4)	0.73
Positive	182 (38.5)	837 (37.6)	
Negative			
Progesterone receptor			
Positive	248 (52.7)	1138 (51.4)	0.61
Negative	223 (47.4)	1077 (48.6)	
HER-2 (IHC)			
0-1+	305 (66.2)	1415 (64.8)	0.57
2+ - 3+	156 (33.8)	770 (35.2)	

[a] Subjects without previous history of cancer (*N*=156), benign breast cancer (*N*=66), previous history of hysterectomy or oophorectomy (*N*=268), and *in situ* breast cancer (*N*=181).
[b] Pearson's χ^2 test
[c] Student's t-test
